# Closed Reduction in a “Hyperextended Supine Position” with Percutaneous Transsacral-Transiliac and Iliosacral Screw Fixation for Denis Zone III Sacral Fractures

**DOI:** 10.1155/2018/6098510

**Published:** 2018-05-23

**Authors:** Hideto Irifune, Suguru Hirayama, Nobuyuki Takahashi, Mitsumasa Chiba, Toshihiko Yamashita

**Affiliations:** ^1^Department of Emergency Medicine Advanced Critical Care and Emergency Center, Sapporo Medical University School of Medicine, Sapporo, Japan; ^2^Department of Orthopedic Surgery, Sapporo Medical University School of Medicine, Sapporo, Japan

## Abstract

**Background:**

Herein, we demonstrate the clinical results of closed reduction in a hyperextended supine position with transsacral-transiliac (TSTI) and iliosacral (IS) screw fixations for Denis zone III sacral fractures.

**Patients and Methods:**

Sixteen consecutive patients with Denis zone III sacral fractures who were treated between January 2009 and September 2016 were evaluated. All patients were treated using percutaneous TSTI/IS screw fixation during closed reduction performed with patients placed in a hyperextended supine position with body manipulation. The clinical and radiological results were evaluated, and the neurological outcomes were retrospectively assessed using Gibbon's classification system. The clinical outcomes were evaluated using the German Multicenter Study Group Pelvic Outcome Scale (POS).

**Results:**

The sacral kyphotic angle was reduced by 18.06° ± 15.26° (mean kyphotic angle: pre-OP, 39.44° ± 20.56°; post-OP, 21.38° ± 7.39°), and fracture translation was reduced by 5.93 ± 4.95 mm (mean fracture translation: pre-OP, 8.69 ± 8.03 mm; post-OP 2.75 ± 3.97 mm). The mean initial Gibbon's score was 3.00 ± 1.32. Among 15 patients with a follow-up duration of over 12 months, the mean reduction loss in the sacral kyphotic angle was 5.87° ± 10.40° and was 1.00 ± 3.00 mm for the fracture translation. The final Gibbon's score was 1.80 ± 1.21, and 73.3% of patients had good results based on the POS score.

**Conclusions:**

Although closed reduction in a hyperextended supine position with percutaneous posterior screw fixation is associated with some surgical limitations and technical difficulties, the procedure is minimally invasive and highly effective for stabilizing Denis zone III sacral fractures.

## 1. Introduction

Denis zone III sacral fractures are generally due to high-energy traumas such as falls from heights, traffic accidents, and crush injuries, with most of these fractures occurring in patients with polytrauma [1]. Zone III sacral fractures are relatively rare injuries, reportedly accounting for only 3–5% of all sacral fractures [2–4]. Importantly, these fractures are associated with a high rate of neurologic injury, including sensory and motor deficits in the lower legs; saddle anesthesia; and bowel, bladder, and sexual dysfunctions [2].

Historically, the standard treatment for Denis zone III sacral fractures did not involve surgery or was limited to sacral laminectomy [1, 5]. However, given the advances in internal fixation techniques, cases with minimal kyphosis and no neurological deficits can be treated using several commonly performed surgical procedures such as spinopelvic fixation [6], posterior plate fixation [7], and iliosacral screw fixation [8]. However, no clear guidelines currently exist regarding appropriate treatment strategies and indications for Denis zone III sacral fractures. Spinopelvic and posterior plate fixation have shown good reduction and neurological recovery but are associated with long operating times. Moreover, the surgery is performed in the prone position, which may be particularly disadvantageous for polytrauma patients. In addition, the use of posterior plates and other spinal instrumentations may cause soft tissue damage and lead to wound complications. To minimize the risk of such complications, minimally invasive approaches have been increasingly used for these fractures. In recent reports, minimally invasive posterior screw fixations for pelvic ring fractures, including transsacral-transiliac (TSTI) and iliosacral (IS) screw fixations, have been reported to have a number of clinical and biomechanical advantages [9–13].

The purpose of the present study was to review our experiences and demonstrate the clinical results of closed reduction in a hyperextended supine position with percutaneous TSTI/IS screw fixation methods for Denis zone III sacral fractures.

## 2. Patients and Methods

Patients with Denis zone III sacral fractures classified as AO/OTA type C pelvic injuries, treated between January 2009 and September 2016, were selected from our trauma database. Patients who had undergone surgical treatment and had been followed up for a minimum of 12 months were selected for analysis. The patients' medical records were reviewed retrospectively, and the fractures were classified by the sacral level according to the method of Roy-Camille (as modified by Strange-Vognsen and Lebech) and the fracture morphology [1, 14, 15].

The following injury data were collected: demographics, mechanism of injury, associated injury, and the ISS. An initial clinical neurological examination was performed if the patient's condition permitted and was graded according to the classification system described by Gibbons et al [5, 16]. Preoperative imaging consisted of pelvic anterior-posterior radiographs and multislice CT scans. Collected postoperative data included the results from clinical and radiological assessments. Radiographic assessments of the sacral kyphotic angle, fracture translation, implant position, and decompression were performed using standard radiography of the pelvis and multislice CT [17, 18]. Additionally, multidisciplinary follow-up examinations were performed by an orthopedic surgeon (H.I.).

### 2.1. Reduction and Operative Techniques

During the 8-year study period, a standardized operative technique using closed reduction and posterior pelvic ring fixations (TSTI and/or IS screw fixation) was performed. In patients with combined sacral fractures and anterior pelvic ring injuries, staged and/or one-stage reconstruction was performed. Injuries of the anterior part of the pelvic ring were treated using an anterior extraperitoneal or percutaneous approach. Posterior pelvic ring fixation was performed through the percutaneous approach.

### 2.2. Operative Procedure

**Figure 1:**
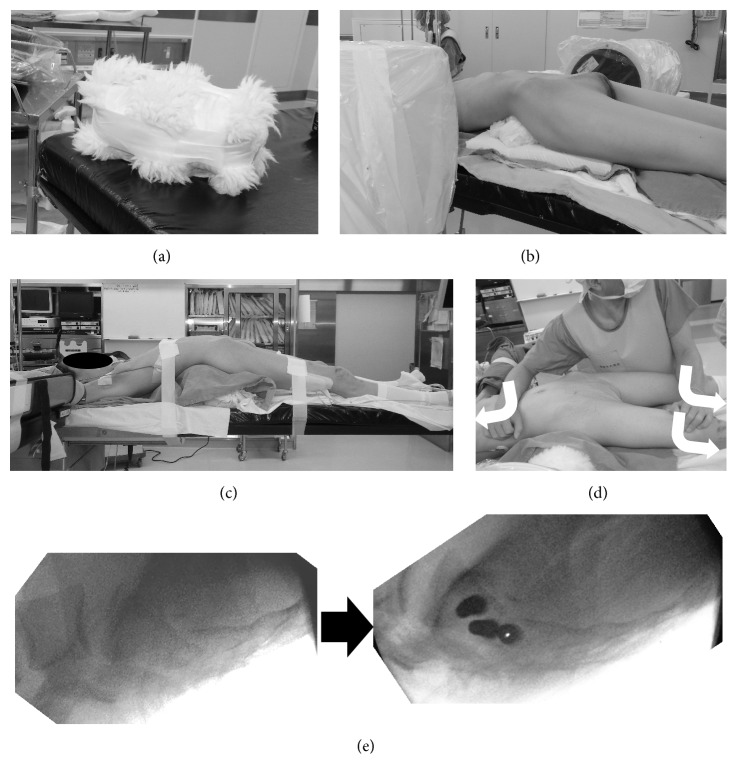
Closed reduction procedure in “hyperextended” supine position with manipulation. (a) A hand-made reduction pillow is shown. (b) The intraoperative position and “hyperextended” supine position is shown. (c) The manipulation maneuver is shown. (d) Intraoperative images are shown. The left image was taken before reduction maneuver and the right image was taken after the maneuvers. If good reduction was obtained, screw insertion was done in the same position. (e) Intraoperative fluoroscopic images pre- and postreduction maneuver. In this case, good reduction was obtained.

For posterior screw fixation, the patients were positioned supine on a radiolucent table and in hyperextension with handmade pillows placed vertical to the sacral fracture line (Figure 1(a)). Under fluoroscopic guidance, closed fracture reduction was performed. When the fracture reduction quality was insufficient, additional pillows were placed and/or manipulation of the patient's body was performed (Figure 1(b)). The manipulation maneuver involved putting a tow in the longitudinal direction while pushing the trunk and lower limbs to the table (Figure 1(c)). After closed reduction, percutaneous screw insertion was performed under fluoroscopic guidance (Figure 1(d)). In principle, two or more TSTI screws were inserted; when this was not possible, the TITS screws were set in combination with an IS screw. Screws utilized at our institute included 6.5 mm (diameter) cannulated cancellous screws provided by Depuy-Synthes, Inc. (partial thread; maximum length: 120 mm) and Meira, Inc. (partial and full thread; maximum length: 150 mm). Direct sacral decompression was not initially performed.

### 2.3. Postoperative Care

The patient was allowed to sit with the torso upright starting on postoperative day 1. Nonweight bearing activities were allowed 4 weeks after definitive surgery. Weight-bearing activities began 5 weeks after definitive surgery.

After definitive pelvic ring fixation, a follow-up CT was performed within 1 week to indirectly evaluate the decompression quality. If a remnant fracture fragment in the sacral canal and/or poor indirect decompression quality was found, a secondary direct sacral decompression was considered. One orthopedic trauma surgeon (H.I.) performed all operative procedures. Early and late complications associated with the surgical treatment were recorded.

### 2.4. Radiological Evaluation

**Figure 2:**
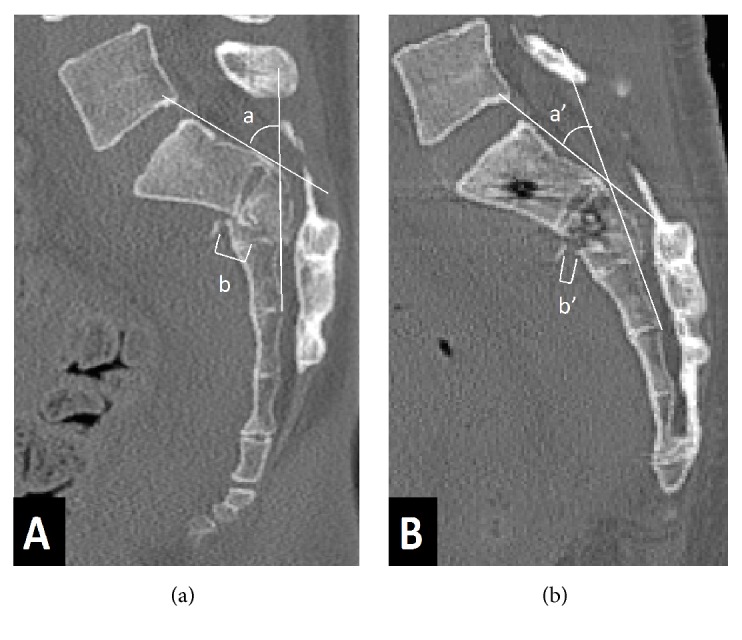
Radiological measurement methods. (a) A preoperative CT scan of the sacrum in the sagittal plane is shown. (b) A postoperative CT scan of the sacrum in the sagittal plane is shown. a and a': sacral kyphotic angle; b and b': sacral fracture translation.

The sacral kyphotic angulation of the Denis zone III sacral fracture was measured from sagittal CT reformations by measuring the angle between the posterior sacral cortices, superior and inferior to the level of the transverse fracture. Fracture translation was also measured from the sagittal CT reformations by measuring the displacement of the anterior cortex of the sacrum above and below the transverse fracture (Figure 2). All measurements involving the preoperative, postoperative, and final radiographs, as well as the CT images, were performed by the first author.

### 2.5. Outcome Evaluation

Lower extremity sensory and motor function and rectal examinations were performed pre- and postoperatively to identify injuries to the lower lumbosacral plexus. Neurological deficits from cauda equina injuries were classified according to the method of Gibbons et al [5]. Improvements in neurological function at the final follow-up were similarly assessed.

The clinical outcomes were evaluated at the final follow-up using the clinical criteria of the German Multicenter Study Group Pelvis Outcome Scale (POS) [19]. These clinical criteria (pain, functional impairment, persistent neurological and urological impairments, and bowel dysfunction) are based on the clinical results and range from 1 to 4 points; a POS score of 3–4 points is considered to be a good outcome, whereas a score of 1–2 points indicates a poor outcome.

### 2.6. Statically Analysis

Data was enrolled through Microsoft Excel 2016 (Microsoft, Redmond, WA), followed by a statistical analysis using IBM SPSS Statistics, version 19 (SPSS, Chicago, IL). Data are given in terms of arithmetic mean and standard deviation. The initial, postoperative, and final follow-up data were analyzed using paired t-test. Data differences were considered significant for values of p < 0.05.

## 3. Results

### 3.1. Patient Demographics (Table 1)

Sixteen patients (6 men, 10 women) with Denis zone III sacral fractures classified as AO/OTA type C pelvic injuries were identified. Table 1 presents the patient demographics, mechanism of injury, ISS, fracture patterns, initial Gibbons' grade, and operative procedures. At the time of injury, the mean age of the patients was 29.50 ± 11.12 years (range: 16–50 years). All patients sustained high-energy traumas. The mechanisms of injury included falls from a height (n = 12; 11 suicidal and 1 accidental), traffic accidents (n = 3), and crush injuries (n = 1).

The mean ISS was 25.94 ± 13.88 (range: 9–50). Thirteen of the 16 patients showed associated injuries, including 9 cases of associated injuries of the head and trunk with an Abbreviated Injury Scale Score ≥3. Other injuries of the spine and extremities were present in 10 patients, while two patients experienced pelvic fracture and urinary tract injuries. At the time of initial examination, neurological deficits were observed in 12 out of 16 patients, with a mean Gibbons' grade of 3.13 ± 1.25 (range: 1–4).

### 3.2. Fracture Types

The transverse fractures involved the following levels of the sacrum: S1 (n=1), S2 (n=7), S2-3 (n=2), S3 (n=3), S3-4 (n=1), S2 + S3-4 (n=1), and S2 + S3 (n=1). The fracture patterns were classified using the Roy-Camille classification system and the fracture morphology. Roy-Camille type 2 fractures occurred in 12 patients and type 3 fractures occurred in 4 patients. Further, H-shaped (n=3), T-shaped (n=4), U-shaped (n=7), and Y-shaped fractures (n=2) were found. A Morel-Lavallée lesion was observed in 1 case, while no cases of open fracture were found.

### 3.3. Operative Treatment (Table 2)

**Figure 3:**
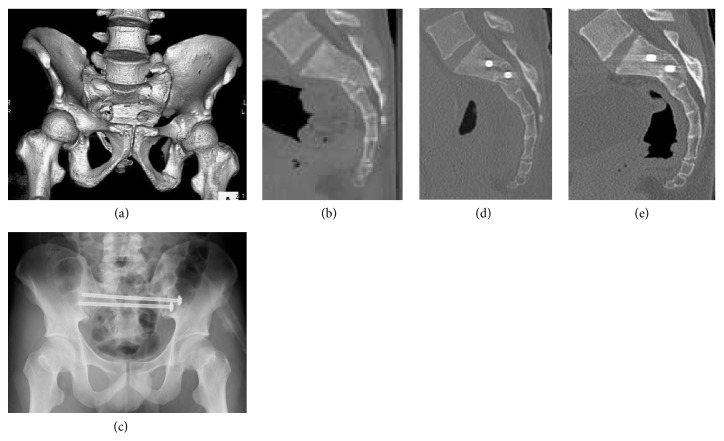
Case 1. A 25-year-old man who was injured from a fall from a height underwent S1 and S2 TSTI partial threaded screw fixation for a Roy-Camille type 2 U-shaped transverse sacral fracture. (a) Preoperative 3-dimensional CT reconstruction of the pelvis is shown. (b) Preoperative CT scan of the sacrum in the sagittal plane is shown (preoperative sacral kyphotic angle: 33°; fracture translation: 10 mm). (c) Postoperative anteroposterior radiograph of the pelvis is shown. (d) Postoperative CT scan of the sacrum in the sagittal plane is shown (postoperative sacral kyphotic angle: 17°; fracture translation: 2 mm). (e) Final follow-up CT scan of the sacrum in the sagittal plane is shown. Slight reduction loss was observed.

**Figure 4:**
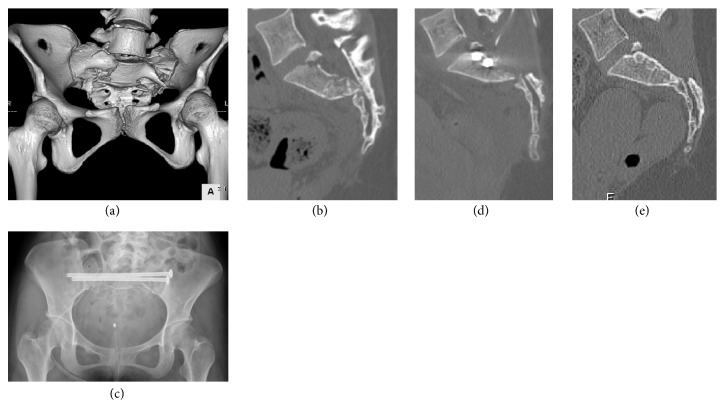
Case 9. An 18-year-old man who was injured from a fall from a height underwent S1 and S2 TSTI full threaded screw fixation for a Roy-Camille type 2 U-shaped transverse sacral fracture. (a) Preoperative 3-dimensional CT reconstruction of the pelvis is shown. (b) Preoperative CT scan of the sacrum in the sagittal plane is shown (preoperative sacral kyphotic angle: 89°; fracture translation: 8 mm). (c) Postoperative anteroposterior radiograph of the pelvis is shown. (d) Postoperative CT scan of the sacrum in the sagittal plane is shown (preoperative sacral kyphotic angle: 32°; fracture translation: 3 mm). (e) Final follow-up CT scan of the sacrum in the sagittal plane is shown. Good reduction quality was maintained.

**Figure 5:**
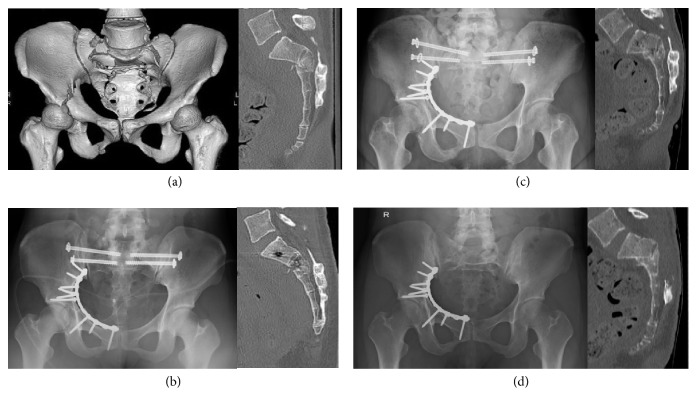
Case 3. This case had the greatest loss of reduction. A 25-year-old woman who was injured from a fall from a height underwent unilateral S1 and S2 bilateral iliosacral partial threaded screw fixation for a Roy-Camille type 2 H-shaped transverse sacral fracture. (a) Preoperative 3-dimensional and sagittal plane CT reconstruction of the pelvis is shown (preoperative sacral kyphotic angle: 63°; fracture translation: 11 mm). (b) Postoperative anteroposterior radiograph and CT scan of the sacrum in the sagittal plane is shown (postoperative sacral kyphotic angle: 28°; fracture translation: 6 mm). (c) The anteroposterior radiograph and CT scan of the sacrum in the sagittal plane taken one month after the initial operative treatment is shown (sacral kyphotic angle: 60°; fracture translation: 14 mm). IS screw loosening was observed. Secondary sacral decompression was performed. (d) Final follow-up anteroposterior radiograph and CT scan of the sacrum in the sagittal plane is shown. Bone healing was achieved, but reduction loss and bladder/bowel dysfunction remained.

Posterior percutaneous internal fixation was performed between 0 and 8 days after the injury (median: 1.31 ± 2.73 days). The operative methods for posterior pelvic ring stabilization included 12 cases of 2- or 3-TSTI screw fixation only (Figures 3 and 4), 1 bilateral S1 IS and S2 TSTI screws fixation, 1 unilateral S1 and S2 IS screw fixations, and 2 bilateral S1 and S2 IS screw fixations (Figure 5). Additional primary fixation was performed in 9 patients, including pubic rami screw fixation (n=4), pubic rami plate fixation (n=1), pubic rami screw/plate and anterior sacroiliac plate fixation (n=1), symphysis plate fixation (n=2), and plate fixations for acetabular fractures (n=2) (Figure 5). The mean operative time for posterior definitive fixation was 64.69 ± 98.65 minutes (range: 10–420 minutes).

Additional operations due to remnant bone fragments and neurological deficits were performed in 5 patients, including delayed direct sacral decompression (n=5). Posterior implant removal was routinely performed in 14 patients after the fractures had healed.

### 3.4. Pre- and Postoperative Radiological Results (Table 2)

The mean preoperative and postoperative sacral kyphotic angles were 39.44° ± 20.57° (range: 13–89°) and 21.38° ± 7.39° (range, 11–36°), respectively. The mean postoperative reduction angle of the sacral kyphosis was 18.06° ± 15.26° (range: 2–57°). The kyphotic angle was improved with a significant difference (p<0.05, 95% confidence interval (CI) 9.93 to 26.19) The mean preoperative and postoperative translations were 8.69 ± 8.03 mm (range: 0–35 mm), 2.75 ± 3.97 mm (range: 0–16 mm), respectively. The mean postoperative translation reduction was 5.93 ± 4.95 mm (range: 0–19 mm). The fracture translation was also improved with a significant difference (p<0.05, 95%CI 3.30 to 8.57).

### 3.5. Radiological, Neurological, and Clinical Outcomes at Final Follow-Up (Table 2)

Fifteen patients were followed for more than 12 months. At the final follow-up (mean: 27.53 ± 19.64 months; range: 12–71 months), the mean sacral kyphotic angle and reduction loss were 27.93° ± 13.01° (range: 13–60°) and 5.87° ± 10.40° (range: -1-32°), respectively. The kyphotic angle was decreased with a significant difference between post-OP and final follow-up (p<0.05, 95%CI -11.62 to -0.11). The mean sacral translation and reduction loss were 3.80 ± 6.71 mm (range: 0-24 mm) and 1.00 ± 3.00 mm (range: -2-8 mm), respectively. The translation was maintained with a no difference (p=0.22, 95%CI -2.66 to 0.66). The mean Gibbons' grade was 1.80 ± 1.21 (range: 1–4) and 8 out of 12 patients (66.71%) with neurological symptoms showed improvement in neurological status. The Gibbons grade was improved with a significant difference (p<0.05, 95%CI 0.65 to 2.02). The mean POS score was 2.93 ± 1.28 (range: 1-4). The clinical results as indicated by the POS score were good and poor in 73.3% (11/15) and 26.7% (4/15) of the patients, respectively (Table 2).

### 3.6. Complications

All patients showed bone union. No case of deep and/or superficial wound infection was noted. Screw loosening was observed in 3 patients; one patient had greater reduction loss (case 3; bilateral S1 and S2 IS screws, see Figure 5). Further, screw malposition was observed in 4 patients; however, in these cases, no new neurological deficits were observed postoperatively.

## 4. Discussion

The present study aimed to show that closed reduction in a hyperextended supine position with manipulations and percutaneous TSTI/IS screw fixation is useful for treating Denis zone III sacral fractures. Currently, the present study is the largest case series regarding closed reduction and percutaneous screw fixation for these fractures [8, 10, 11, 13]. Furthermore, the present study includes the greatest number of cases involving the supine position maneuver.

Denis zone III sacral fractures may be H-, U-, T-, or Y-shaped [20], and sacral dislocations may be anterior or posterior [1]. When treating these fractures, it is important to reduce the sacral kyphotic angle fracture translation and to maintain this reduced position. Previously, the outcomes of several treatments for these fractures have been reported. Siebler et al. reported the results of conservative treatment [21], and found that the sacral kyphotic angle increased by 4.1° (from 36.4° to 40.5°) posttreatment. In terms of operative treatment, Schildhauer et al., Tan et al., and Lindahl et al. reported that using spinopelvic fixation, the initial kyphotic angles of 43°, 32°, and 38° were improved to the final angles of 21°, 19°, and 22°, respectively [6, 20, 22]. Nork et al. reported that, after using IS screw fixation, the kyphotic angle was reduced from 29.2° to 28.2° [8], whereas König et al. reported that, after using TSTI screw fixation in 3 patients, the sacrococcygeal and pelvic incidence angles were reduced from 84° and 75° preoperatively to 58° and 56° postoperatively, respectively (reduction losses of 14° and 15°, respectively) [10]. Ruatti et al. reported that, in a supine position, hyperlordosis, skeletal traction, and percutaneous IS screw fixation had good reduction results in 3 cases [13]. In the present study, the pre- and postoperative kyphotic angles were 40° and 22°, respectively, indicating a reduction loss of 4.9°. In terms of translation, Lindahl et al. reported that the mean pre- and postoperative translations with spinopelvic fixation were 15.5 and 5.8 mm, respectively [22], whereas in the present study, the corresponding values were 8.9 and 2.8 mm. In the present study, one patient with IS fixation only (Case 3) was observed to have a high reduction loss and screw loosening. Taken together, these clinical data suggest that TSTI screw fixation is equal to spinopelvic fixation. Furthermore, Min et al. reported that 2-TSTI screw fixation was superior to spinopelvic fixation in a zone 2 sacral fracture model from a biomechanical viewpoint [23]. Therefore, we believe that TSTI screw fixation in the supine position is an effective, rigid, and minimally invasive procedure for Denis zone III sacral fracture fixation.

Denis zone III sacral fractures have been reported to result in neurological injury of varying severity in up to 100% of patients [6–8, 20–22, 24]. Accordingly, in the present study, neurological deficits were seen in 12 of 16 patients (75%). However, neurological recovery has been shown to occur in 50–100% of patients with these fractures [6–8, 10, 20, 21]. Early surgical treatment of the sacrum, including restoration of spinopelvic stability and decompression of the nerve roots, indirectly or directly, is thought to provide the best possible environment for neurological recovery. Schildhauer et al. reported that neurological recovery depends on the extent of nerve damage at the time of injury [6]. However, Siebler et al., in their study of nonoperative treatment, reported that the recovery rate was still as high as 85.7%. [21] Thus, the best treatment method, in terms of neurological outcomes, remains unclear. In the present study, the overall neurological recovery rate was 66.7% (8/12). A lack of neurological recovery was observed in four cases (cases 3, 4, 12, and 15). Of these, one case (case 4) involved a spinal cord injury merger. In an additional case (cases 12), decompression of the postoperative sacral canal was deemed sufficient; however, no neurological recovery was observed. In cases 3 and 15, because of a suspected reduction loss in the kyphotic angle and translation, additional nerve decompression surgery was performed (case 3); however, nerve recovery could not be obtained. In addition, four cases (cases 6, 7, 8, and 9; see Figure 4) underwent additional decompression because of remnant fragment and/or insufficient neurological recovery and were finally considered to have sufficient neurological recovery. Based on our experience and the report by Schildhauer et al. [6], the likelihood of neurological recovery appears to be dependent on the degree of nerve damage at the time of injury. However, for maximum neurological recovery, we consider indirect or direct nerve decompression to be necessary. Hence, in our treatment strategy, indirect reduction and decompression are performed in the acute phase and if the postoperative CT shows incomplete decompression and/or remnant fracture fragments in the sacral canal, secondary direct sacral decompression is consequently performed.

We consider TSTI/IS screw fixation to be a less invasive method, associated with a relatively low complication rate. In our series, the mean operative time was approximately 65 minutes. In cases involving TSTI or IS screw insertion only, the screw insertion time was approximately 10 minutes per screw. In addition, the time required for our preoperative closed reduction procedure is about 20 to 30 minutes. Hence, in cases requiring only closed reduction and screw fixation, patients can be treated in the acute phase. Furthermore, in cases involving screw fixation only, the patient is positioned supine, which is often the most comfortable position for polytrauma patients. In addition, TSTI/IS screw fixation is associated with a very low profile and few surgical site complications. In fact, in our study, no surgical site complications were observed. In past reports using different approaches, the surgical site complication rate was as high as 38% [6, 7, 20]. Spinopelvic fixation is associated with a particularly high complication rate in pelvic ring fracture treatment [6, 20], and posterior soft tissue complications are associated with poorer outcomes.

There are several limitations to the present study. First, the study population was relatively small and did not allow for a highly powered statistical analysis; therefore, our conclusions should be interpreted with caution. Second, there are operative limitations for TSTI/IS screw fixation. In TSTI/IS screw fixation for zone III sacral fractures, at least two screw insertions (2 TSTI screws, or 1 TSTI screw and 1 IS screw) are required, as the screws need to provide resistance against the vertical load shear force and the rotational force of the sacral vertebral body. However, it has been reported that ilio-sacro-iliacal corridors for intraosseous implants were not inserted in 18–25% and 10–12% of S1 and S2 cases, respectively; moreover, a high frequency of this issue has been reported for female patients [25–27]. Thus, owing to anatomical variance and different fracture types, this method is not always useful.

In conclusion, the current study showed that closed reduction in a hyperextended supine position with TSTI/IS screw fixation is effective for treating Denis zone III sacral fractures in terms of the fracture reduction, loss of reduction, neurological recovery, and clinical outcomes. Thus, despite some limitations, we believe that our procedure is an effective and appropriate method for treating Denis zone III sacral fractures.

## Figures and Tables

**Table 1:** Preoperative clinical data and operative procedures.

Patients No.	Age/Sex	Mechanism of Injury	Level of transverse fracture	Classifications		ISS	Day of OP	OP procedure	
				R-C	Description			Anterior	Posterior
1	25/M	FOH	S2	2	U	34	0		S1; TSTI(P), S2; TSTI(P)
2	20/M	FOH	S2	2	H	22	0	PR screw	S1; IS×2(P) S2; IS×2(P)
3	25/F	FOH	S2	2	H	29	0	acetabulum plate	S1; IS×2(P) S2; IS×2(P)
4	40/F	FOH	S2/3	2	T	43	0		S1; TSTI(P) S2; TSTI(P)
5	16/F	WTA	S3	2	Y	17	7	PR screw×2	S1; TSTI(P) S2; TSTI(P)
6	21/M	FOH	S2	2	T	13	1		S1; TSTI(F) S2; TSTI(F)
7	29/F	FOH	S2	2	U	10	0		S1; TSTI×2(F) S2; TSTI(F)
8	20/M	CR	S3/4	2	Y	35	8	PR screw, PR plate	S1; TSTI(F) S2; TSTI(F)
9	18/F	FOH	S3	2	U	50	0		S1; TSTI(F) S2; TSTI(F)
10	25/F	FOH	S3	2	H	34	0	acetabulum plate	S1; TSTI(F) S2; TSTI(F)
11	29/M	FOH	S2	3	U	9	0		S1; IS×2(P) S2; TSTI(F)
12	23/F	FOH	S2, S3/4	2	U	16	0		S1; TSTI×2(F) S2; TSTI(F)
13	50/M	FOH	S2/3	3	T	25	5	symphysis plate	S1; IS(P) S2; TSTI(F)
14	39/F	WTA	S2, S3	2	T	10	0	PR screw×2	S1; TSTI(F) S2; TSTI(F)
15	49/F	FOH	S1	3	U	50	0	symphysis plate	S1; TSTI×2(F)
16	43/F	CTA	S2	3	U	18	0	PR screw×2	S1; TSTI(F) S2; TSTI(F)

ISS: Injury Severity Score; R-C: Roy-Camille; OP: operative; TSTI: transsacral-transiliac screw; IS: iliosacral screw; U: U-shaped; H: H-shaped; T: T-shaped; FOH: fall from height; CR: crush injury; TA: traffic accident; F: female; M: male; P: partial threads screw; F: full thread screw.

**Table 2:** Radiological and clinical findings.

Patient No.	Kyphotic angle			Fracture translation			Gibbons		POS clinical	FU month
	Initial	post-OP	Final	Initial	Post-OP	Final	Initial	Final		
1	33	17	20	10	2	3	1	1	4	12
2	50	36	35	12	0	0	4	1	4	24
3	63	28	60	11	6	14	4	4	1	71
4	24	16	16	0	0	0	4	4	1	64
5	31	22	22	9	4	4	4	1	4	14
6	46	31	30	5	2	0	4	2	3	18
7	65	25	36	12	0	0	4	2	3	12
8	21	13	13	3	0	0	3	1	4	48
9	89	32	32	8	3	1	4	2	3	32
10	27	15	15	11	3	3	3	1	4	19
11	32	21	24	5	0	0	1	1	4	15
12	58	23	40	35	16	24	4	4	1	40
13	13	11	-	4	2	-	1	-	-	2
14	31	21	21	0	0	0	2	1	3	12
15	28	17	41	8	4	6	4	4	1	20
16	20	14	14	6	2	2	1	1	4	17

## Data Availability

The data used to support the findings of this study are available from the corresponding author upon request.
